# A millennium of increasing diversity of ecosystems until the mid‐20th century

**DOI:** 10.1111/gcb.16335

**Published:** 2022-07-22

**Authors:** Inês S. Martins, Maria Dornelas, Mark Vellend, Chris D. Thomas

**Affiliations:** ^1^ Department of Biology University of York York UK; ^2^ Leverhulme Centre for Anthropocene Biodiversity, Berrick Saul Second Floor University of York York UK; ^3^ Centre for Biological Diversity, School of Biology University of St Andrews St Andrews UK; ^4^ Département de Biologie Université de Sherbrooke Sherbrooke Quebec Canada

**Keywords:** Anthropocene, diversity metrics, ecosystem diversity, global change, land‐use change, spatial ecology, spatio‐temporal

## Abstract

Land‐use change is widely regarded as a simplifying and homogenising force in nature. In contrast, analysing global land‐use reconstructions from the 10th to 20th centuries, we found progressive increases in the number, evenness, and diversity of ecosystems (including human‐modified land‐use types) present across most of the Earth's land surface. Ecosystem diversity increased more rapidly after ~1700 CE, then slowed or slightly declined (depending on the metric) following the mid‐20th century acceleration of human impacts. The results also reveal increasing spatial differentiation, rather than homogenisation, in both the presence‐absence and area‐coverage of different ecosystem types at sub‐global scales—at least, prior to the mid‐20th century. Nonetheless, geographic homogenization was revealed for a subset of analyses at a global scale, reflecting the now‐global presence of certain human‐modified ecosystem types. Our results suggest that, while human land‐use changes have caused declines in relatively undisturbed or “primary” ecosystem types, they have also driven increases in ecosystem diversity over the last millennium.

## Graphical abstract

Land‐use change is widely regarded as a simplifying and homogenising force in nature. However, we found progressive increases in the number, evenness, and diversity of ecosystems present across most of the Earth's land surface until the mid‐20th century acceleration of human impacts. Nonetheless, geographic homogenization was revealed for a subset of analyses at a global scale, reflecting the now‐global presence of certain human‐modified ecosystem types. While the global story of biodiversity change involves the loss and decline of certain ecosystem types, the full story is more complex and interesting, involving gains and increases in other ecosystem types, and increased ecosystem diversity at most spatial and temporal scales.

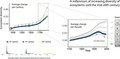

## 1 INTRODUCTION

Humans have been reshaping the processes, structure, and biological composition of ecosystems for millennia (Ellis, 2021; Mottl et al., 2021). These changes are typically regarded as the most important proximate drivers of terrestrial biodiversity change: the International Union for Conservation of Nature (IUCN) lists various aspects of land‐use change and altered management as eight of the top 10 threats to species (IUCN, 2018), while the Intergovernmental Science‐Policy Platform on Biodiversity and Ecosystem Services (IPBES) identifies “changes in land and sea use” as the largest driver of “changes in nature” (IPBES, 2019). These changes are recognised by the United Nations Convention on Biological Diversity (CBD), which has ecosystem extent at the core of its post‐2020 agenda (CBD, 2020).

The CBD recognises biodiversity as encompassing the “diversity within species, between species and of ecosystems” (CBD, 1992), and hence the diversity of ecosystems is regarded both as a key component of biodiversity in its own right, as well as a key determinant of species richness (Stein et al., 2014). The CBD then defines an ecosystem as “a dynamic complex of plant, animal, and micro‐organism communities and their non‐living environment interacting as a functional unit.” Almost all attention in the literature has been on changes in the area and coverage of specific ecosystem types, with considerable concern about the extent and rate of loss of relatively unmodified (sometimes referred to as “primary”) ecosystem types throughout the world (Brauman et al., 2019). In contrast, little information is available on temporal changes in ecosystem diversity per se. Because ecosystem diversity is one of the three key elements of CBD's definition of biodiversity, this is a glaring knowledge gap.

Ecosystems also support other elements of biodiversity, with local species richness typically higher in primary vegetation than in modified ecosystems (Newbold et al., 2015). However, because human‐modified ecosystems often contain sets of species that differ from those in the primary ecosystem (e.g., there may be limited compositional overlap between species in derived pastures and those in an “original” primary forest; Newbold et al., 2015), it is unclear how mosaics of primary and modified ecosystem types might affect species diversity at landscape and regional scales. In Ontario, Canada, Desrochers et al. (2011) showed that bird diversity (species richness) increased with ecosystem (land‐cover) diversity at a landscape scale and that landscapes containing a mixture of primary and derived ecosystems contained more species in total than landscapes dominated by primary ecosystems, and more than in landscapes entirely covered by human‐modified ecosystems. Thus, establishing how ecosystem diversity has changed at landscape and regional scales is critical if we are to understand global patterns of species diversity.

Despite the importance of ecosystem diversity changes in their own right and as a determinant of regional species diversity, there is no comprehensive analysis of how land‐use change has altered the diversity of ecosystem types over time and space. It is unclear whether land‐use change has generally led to landscape simplification (e.g., as in some extensive arable landscapes) or landscape diversification (a greater mixture of ecosystems). Ecosystem diversity and its changes over time represent a major gap in our broader understanding of human impacts on biodiversity.

## 2 MATERIAL AND METHODS

### 2.1 Ecosystems

Ecosystems are inherently dynamic and complex, such that any categorization is unavoidably a gross oversimplification. Yet such simplification is needed if we hope to understand global‐scale changes (Ellis & Ramankutty, 2008). Ecosystems as defined by the CBD are difficult to delimit unambiguously, overlapping in some contexts with concepts such as “biomes,” “communities” and “habitats.” Here, we designate land cover types that contain distinct plant‐based physical structures and their associated biotas as “ecosystems.” For example, primary forest (natural), rangelands (semi‐natural), and arable (anthropogenic) land covers are all included within this definition, and we regard a landscape that contains all three as having greater ecosystem diversity than those that only contain one of them (see Section 2). Thus, our use of the term “ecosystem diversity” is equivalent to most uses of the terms “habitat diversity,” “habitat heterogeneity,” and “landscape heterogeneity,” encompassing the variety of major vegetation types in a specified area or region.

### 2.2 Land‐use data

We consider the last millennium, given the antiquity of many land‐use changes, and we explore changes at a global scale to avoid the risk of selecting unrepresentative regions. Of candidate data sets of sufficient duration (including Kaplan & Krumhardt, 2011; Klein Goldewijk et al., 2017; Stephens et al., 2019; Hurtt et al., 2020), only the newly released Land Use Harmonization version 2 (LUH2) data set (http://luh.umd.edu/data.shtml) had sufficient spatial resolution, temporal resolution *and* land‐use thematic resolution for us to be able to perform the analyses. We downloaded the LUH2 global annual gridded maps (0.25° × 0.25° cell resolution) that provide the fraction of each of 12 land‐use types in each cell for historical land‐use change (from 900 to 2000, the 11 full century‐long periods within the database). The 12 land‐use categories were as follows: forested primary vegetation, non‐forested primary vegetation, forested secondary vegetation, non‐forested secondary vegetation, managed pasture, rangeland, urban land, plus five functional crop categories (including plantations). To keep in line with our structural definition of ecosystem types, we grouped the crop land‐use data into two major anthropogenic ecosystem types, cropland and tree plantations, resulting in a final set of nine anthropogenic and relatively natural ecosystems (Figure S1); similar categorization as recently used in Chapter 4 of the IPBES global assessment (Shin et al., 2019). Still, we recognize that this is a simplistic categorization of terrestrial ecosystems. Therefore, to test the robustness of our conclusions to the method of categorizing ecosystems, we created two, more finely divided classifications of ecosystems where different assumptions were made. In the first alternative classification, we assume that natural ecosystems—primary and secondary (forested and non‐forested)—are distinct across major bioclimatic regions, while anthropogenic ecosystems are not. In the second alternative classification, we assume that *both* natural and anthropogenic ecosystem types vary between major bioclimatic regions. “Biological reality” is likely to fall somewhere in between these two assumptions (e.g., arable crops may be somewhat more biologically similar in different bioclimate zones than are natural ecosystems, but they will not be exactly the same), so this represents a sensitivity evaluation.

The 14 bioclimatic regions were defined by the global WWF's biomes map, and ecosystem types in each cell were reclassified based on their spatial location, after overlapping both types of maps (i.e., biome map and LUH2 annual gridded maps). Under the first alternative classification, only natural ecosystems were reclassified. For example, primary forest in cells across boreal regions was considered to be a different ecosystem from primary forest in cells across temperate regions, but pastures were considered to be the same ecosystem independently of their location across the globe, leading to a total of 61 distinct ecosystem types (56 relatively natural ecosystems and 5 anthropogenic ecosystem types). Under the second alternative classification all ecosystems were reclassified, leading to 126 distinct ecosystem types (56 relatively natural ecosystems and 98 anthropogenic ecosystem types). Because the structure, biological composition and potential fates of ecosystems vary geographically, we carried out separate analyses for different sub‐regions, given by each of 17 IPBES sub‐regions and each of 14 WWF biomes (and also tested for effects of spatial scale, see below), in addition to a global analysis of ecosystem types. Together, these sub‐global analyses test for the robustness of the results to geographic region, and to the definition of ecosystem (i.e., ecosystem type × region combinations effectively represent a narrower definition of ecosystem type).

In our analysis, we considered cells across all regions of the world, but removed 8114 cells on the boundaries of WWF Biomes (i.e., cells that cross two or more biomes after overlap: ~4.7% of the cells), since the precise locations of such boundaries are not static (their distribution is tightly linked to dynamic climatic and geological processes), and likely to have changed over the last millennium (Moncrieff et al., 2016). Finally, because the LUH2 data set was built on a quarter degree grid cell format (i.e., 0.25° grid cell resolution), there are fewer geographic grid cells per unit of area of land (i.e., each cell is larger) at the equator than at higher latitudes. As this could lead to geographical bias, all LUH2 global annual gridded maps were re‐gridded to an equal‐area global grid build using an equal‐area projection, where each cell has ~769 km^2^, or ~27.8 km × 27.6 km at the equator (equivalent to a 0.25° grid cell resolution at the equator; Figure S2). During re‐gridding, all within‐cell original ecosystem areas were recalculated (based on spatial overlap area) to match this new resolution (i.e., 0.25°‐equivalent grid), resulting in a final data set containing 173,892 cells. For each year, presence (a given ecosystem is present, 1, or absent, 0, in each cell), area (total area of the ecosystem in the cell) and coverage (i.e., fraction of the land area of a cell occupied by a given ecosystem, thereby adjusting for part‐land cells) of each ecosystem were calculated for each equal‐area grid cell (Figure S1). These were averaged at different spatial scales (grain and extent) for each of the three ecosystem classifications: 9 (original classification), 61 (alternative classification 1), or 126 ecosystem types (alternative classification 2). All statistical analyses were performed in R‐3.6.3 (RC Team, 2019).

### 2.3 Ecosystem diversity metrics (*α* diversity)

Ecosystem α diversity represents the variety of ecosystem types in a cell of a given size. We quantified changes to the diversity of ecosystems within grid cells at different times and scales using five complementary metrics. The metrics we use are equivalent to those typically applied to measure species diversity, but with different ecosystems in place of different species, and the area of an ecosystem used in place of the abundance of a given species. These are metrics of: richness (the number of ecosystems per cell), evenness (balance of ecosystem types), heterogeneity (number and relative area of ecosystems), and composition (number, relative area and the compositional distance between ecosystems). Specifically, within‐cell richness was calculated as the number of unique ecosystem types present at a given grid cell. Evenness estimates were computed using Pielou's evenness index (*J*) and heterogeneity using both the Shannon diversity index (*H*′) and Simpson diversity index (*D*). Pielou's evenness index (*J*) measures the extent to which the area of two or more ecosystems are similar (calculable for all cells containing two or more ecosystem types), and increases with increased evenness, where 0≤J≤1. Shannon diversity index (*H*′) takes into consideration both the number of ecosystems present, and the area of each, thus jointly reflecting the two major contributions to diversity (the number of ecosystem types and the area of each), increasing with increased diversity. Simpson diversity index (*D*) is similar to Shannon, however it gives more weight to common or dominant ecosystems. These three indices were computed using R (“vegan” package). Together, these four metrics describe the diversity of ecosystems.

The number of species that can be accommodated within a region (grid cell) can depend on the distinctiveness of the biota associated with each ecosystem type (i.e., dissimilarity in species composition between ecosystems), in addition to the number ecosystems present, and the area of each. Therefore, our fifth metric of within‐cell ecosystem α diversity is Rao's quadratic entropy index (Rao*Q*, where 0≤RaoQ≤1), where we incorporated all three aspects by considering the expected biotic dissimilarities among ecosystems (using a compositional distance matrix for species dissimilarities between ecosystem types), weighted by the area of each ecosystem type. The use of Rao*Q* is important because if, for example, all human‐derived land use types contained the same (small) subset of generalist species, Rao*Q* would be low in a mixed‐use landscape, but if each land use contained at least some specialist species that did not occur in others Rao*Q* estimates would be higher. This index can be understood as the mean pairwise distance (as defined by their composition) among ecosystems weighted by ecosystem area. If $p_{i}$ and $p_{j}$ are the proportional area of ecosystem i and j within a cell, respectively, and the mean dissimilarity between ecosystems i and j is $d_{\mathrm{ij}}$, then the Rao coefficient has the form:

$$\mathrm{Rao}Q=\sum_{i=1}^{E}\sum_{j=1}^{E}d_{\mathrm{ij}}p_{i}p_{j},$$

where *E* is the number of ecosystems in the cell and $d_{\mathrm{ij}}$ varies from 0 (two ecosystems have exactly the same species composition) and 1 (two ecosystems have completely different species). If $d_{\mathrm{ij}}$ = 1 for any pair of ecosystems (so each pair of ecosystems is completely different in terms of their species), then Rao*Q* is expected to be equivalent to Simpson's diversity index. For this analysis, we use a compositional dissimilarity matrix derived from Newbold et al. (2015), who reported empirical differences in species compositions within and between ecosystem types (1 − Sørensen index). However, not all ecosystems sampled by Newbold et al. (2015) were a perfect match to the ones defined in this study, based on the original descriptions of the ecosystems. As such, some assumptions were needed to allocate or adapt some of the dissimilarity values reported by Newbold to our analysis. For instance, Newbold et al. (2015) did not distinguish between non‐forested and forested primary land, as such we use their reported $d_{\mathrm{ij}}$ for “primary vegetation” for both ecosystems (ultimately assuming that non‐forested and forested primary land have similar species compositions, and differences to the other ecosystems). A similar assumption was used to retrieve $d_{\mathrm{ij}}$ values for managed pastures and rangelands, as Newbold et al. (2015) considered both ecosystems as “pastures” (based on descriptions). Finally, $d_{\mathrm{ij}}$ for non‐forest secondary land was given by the average of the $d_{\mathrm{ij}}$ reported for “intermediate secondary vegetation” and “young secondary vegetation,” while forested secondary vegetation matched the “mature secondary vegetation” description as reported by Newbold et al. (2015), and no further processing was necessary. Note that we used values rescaled relative to the maximum $d_{\mathrm{ij}}$ reported, instead of rescaled relative to comparisons between two primary vegetation communities (as presented by Newbold et al., 2015 in their figures). The final compositional distance matrix used in our analysis can be found in the Zenodo repository (see “Data and Code Availability Statement”). Rao*Q* estimates were then computed using the function *rao.diversity* in R (“SYNCSA”) package.

### 2.4 Temporal trends in *α* diversity

The LUH2 data layers were built on multiple model inputs (e.g., using existing local/regional level land statistics or records, population and cultural reconstructions, historical maps), with uncertainty expected to be highest in the distant past. While these data are reported as annual values, data for years within centuries up to 1700, and years within decades from 1700 to 2000, were not independent of one another; they reflected the original temporal resolution of the underlying land‐use data before and after 1700 (see Figure S3 for more details and visual representations). Thus, to enable temporally consistent comparisons, we first generated two time‐series for each metric: within‐cell centurial means for 900 to 2000 (100‐year windows), and within‐cell decadal means for 1700 to 2000 (10‐year windows), when data quality improved. For example, for the first centurial time‐period (10th century), the 100‐year window would be the years 900–999, and within‐cell α diversity estimates will be the average of all 100 yearly estimates for that same grid cell. These averages were then represented values appropriate to the midpoint of the time period, in this instance 950. Similarly, for the first decadal time‐period, the 10‐year window would be the years 1700–1709, and within‐cell α diversity estimates will be the average of all 10 yearly estimates for that same grid cell, and linked in analyses to the midpoint of that decade.

**FIGURE 1:**
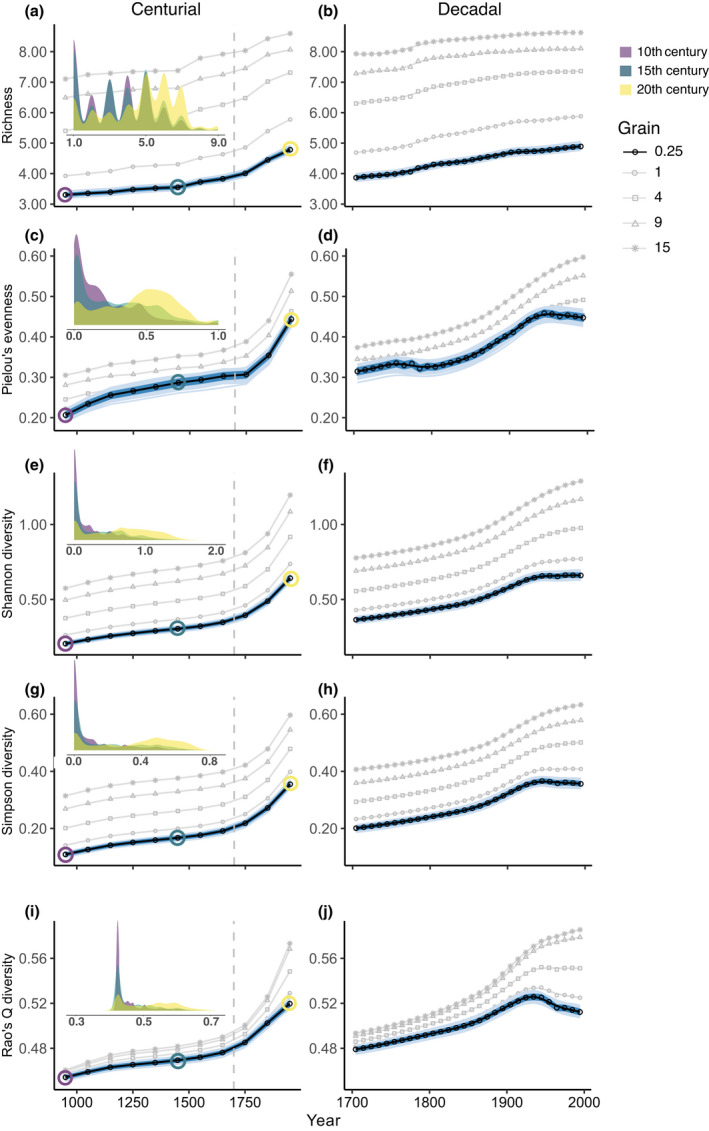
Levels of local ecosystem diversity from 900 to 2000. (a, b) within‐cell ecosystem richness (mean numbers of ecosystem types per cell), (c, d) Pielou's evenness, (e, f) Shannon diversity index, (g, h) Simpson diversity index, and (i, j) Rao's quadratic entropy index. (c, d) and (i, j) scale from 0 (minimum) to 1 (maximum evenness and diversity). The left‐hand graphs show the centurial trends (from 900 to 2000), where each point on the graph represents the spatially‐averaged means across all equal‐area cells found over a 100‐year period plotted on the mid‐point of the century, while the right‐hand graphs show the decadal averages (from 1700 to 2000) plotted on the mid‐point of the decade. Solid lines represent the smoothed trends (cubic regression splines). Four additional grains of analysis (equivalent to the areas of a 1°, 4°, 9° and 15° grid cells at the equator) are shown in gray (larger grid cells typically contain more ecosystem types, and hence usually have higher diversity values). For the 0.25°‐ equivalent scale (lowest resolution), the variability in the global means is shown by the individual global mean trends of 1000 draws (each draw contained 1730 random sampling cells—1000 light blue smoothed lines, giving the appearance of pale blue shading around the trend) together with its interdecile range (darker blue shading, where the upper and lower bounds are given by first and ninth deciles, respectively, at each time‐period). Inset density plots on the left show the distribution of the individual cell estimates of the different metrics at 3 points in time.

**FIGURE 2:**
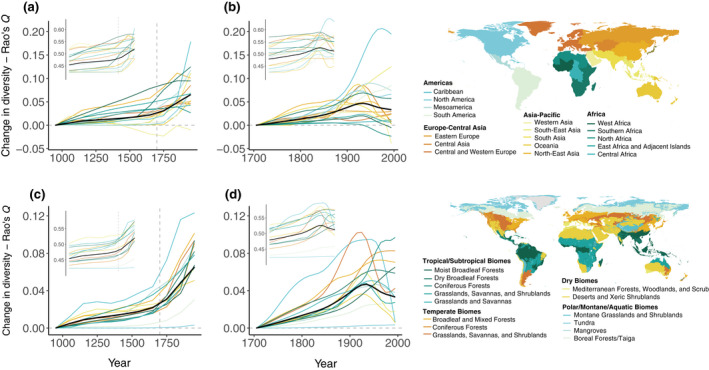
Regional changes in local ecosystem diversity (α‐diversity). Main plots show net change in local ecosystem diversity for (a, b) each IPBES sub‐region and (c, d) each WWF Biome, as measured by Rao's *Q* index. Change is measured relative to the Rao index value at the start of each time‐series. Centurial trends (a, c) are shown relative to the 10th century, and decadal trends (b, d) relative to first decade of 1700. Insets show absolute values. Colours represent different regions. Black lines show the global trends. All continuous lines are smoothing splines applied through the average estimates for a given region. Points are omitted for sake of simplicity, but see Figures S5 and S6 for more details, including trends for other diversity metrics.

In addition to absolute diversity in time estimates (as in Figure 1), we also calculate within‐cell net change (as the difference to the baseline) for each metric (as in Figure 2). Temporal baselines were given by the first time‐period of each time‐series (10th century and first decade of the 1700s, for the centurial and decadal time‐series, respectively). Such baselines should not be regarded as “pristine,” but represent states where earlier human‐induced shifts have already occurred (Kopf et al., 2015). Changes since those times represent subsequent deviations.

For each time‐period, absolute and net‐change grid‐based estimates for each metric were spatially averaged across the globe, and separately averaged for broad biogeographic regions (IPBES sub‐regions and WWF biomes). Spatial averages were weighted by the land‐use area of grid cells (because of land/water cover). All global and regional estimates (including mean, SE, SD, CIs, min, max, quantiles) can be found in Table S2. Global absolute mean values are shown in Figure 1 and net‐change mean values in Figures S4 and S5, while both absolute and net‐change regional mean values are shown in Figure 2 and Figures S6 and S7 (for IPBES sub‐regions and WWF biomes, respectively). In all figures, we additionally fitted smooth lines (cubic regression splines) to the time‐period average estimates, to better visualize the temporal trends in α diversity.Bootstrapping is a common statistical technique for estimating the accuracy of an estimator and its statistical significance. Here, we visualized the variability in the global and regional means by drawing 1000 random samples of n cells from our grid data set $\left(x_{1}\ldots \;,\;x_{n}\right)$, proportionally distributed across all IPBES sub‐regions (to avoid over/under‐sampling regions), and calculating for each time‐period and metric the i sample means $\left(M_{1}\ldots \;,\;M_{i}\right)$ across cells, where $M=f\left(x_{1}\ldots \;,\;x_{n}\right)$. For all draws, sample n was 1730 cells, which represents 1% of the total number of cells. This n allows us to have a rich enough sample to represent (describe) the population, but small enough to avoid significant duplication (on average, only 21 cells were shared between pairs of draws) and most spatial‐autocorrelation issues. Individual 1000 sample estimates (mean, SE, SD, CIs, min, max, quantiles) can be found in Table S3, and mean variability estimates (mean, SE, SD, CIs, min, max, quantiles) across the 1000 samples can be found in Table S4. The individual 1000 sample means are also shown in Figure 1, where each individual light blue line (giving the appearance of pale blue shading in Figure 1) is given by a cubic smoothing spline applied throughout the average estimates for a given sample. The interdecile range of all 1000 different sample means (i.e., area where 80% of all means fall) is shown in both Figure 1 and Figures S6 and S7, where the upper and lower bounds are given by the first and ninth deciles of all 1000 means across each time‐period.

### 2.5 Ecosystem diversity metrics (*β*‐diversity)

We measured β‐diversity as the dissimilarity among pairs of cells using two indices: an incidence‐based index (Jaccard dissimilarity index) and an abundance‐based index (Bray–Curtis dissimilarity index), again extending species‐level diversity metrics to ecosystems. The Jaccard index, as applied here, estimates the extent to which any pair of locations (grid cells) share ecosystem types (0 for exactly the same ecosystems present to 1 for no overlap in the ecosystem types present). We used the “betapart” package in R to calculate and decompose the incidence‐based dissimilarity metric (Jaccard‐$\beta_{\mathrm{jac}}$) into turnover ($\beta_{\mathrm{jtu}}$) and nestedness ($\beta_{\mathrm{jne}}$) components.

The Bray–Curtis ($\beta_{\mathrm{bc}}$) dissimilarity metric takes into account differences in the area of each ecosystem type between pairs of cells, as well as differences in the identities of each ecosystem type; decomposed into balanced variation ($\beta_{\mathrm{bc}-\mathrm{bal}}$ − the areas of some ecosystems decline and other ecosystems increase from one cell to another) and any abundance gradient ($\beta_{\mathrm{bc}-\mathrm{gra}}$ − the areas of all ecosystems decline or increase equally from one cell to the other; Baselga, 2017). Bray–Curtis values also vary from 0 (two cells are identical in which ecosystems are present and in the areas of each ecosystem) and 1 (no ecosystems in common).

### 2.6 Temporal trends in spatial β‐diversity of ecosystems

We characterized spatial β diversity change as the average pairwise dissimilarity of ecosystem composition between pairs of grid‐cells. Average pairwise dissimilarity is known to be a robust measure of spatial heterogeneity because it estimates the expected difference between a random pair of sites (Marion et al., 2017). The spatial scaling of β diversity is also important because the metrics indicate ecosystem differences between locations. Therefore, we again varied the grain of analysis (size of the cells), and how dissimilarity changes when we increase the possible distance between cells (extent), which we varied by sampling pairs of smaller cells (0.25°‐, 1°‐, 9°‐equivalent) within larger cell areas, and up to global extent.

For each combination of grain and extent, we calculated century‐ and decadal‐long spatial dissimilarity values between pairs of the smaller cells. Then, for each centurial/decadal time‐period, global average pairwise dissimilarity was calculated by averaging all pairwise comparisons estimates:

$$\bar{\beta \left(\Omega ,\Psi \right)}=\frac{\sum_{i}p_{k,i^{\Psi }}^{\Omega }}{N\left(p_{i^{\Psi }}^{\Omega }\right)}$$

where $p_{k,i^{\Psi }}^{\Omega }$ is the mean dissimilarity between the pair of cells *i* (of grain size Ψ) in a given time‐period, in the spatial sampling window *k* of size Ω (extent), and $N\left(p_{i^{\Psi }}^{\Omega }\right)$ is the total number of pairwise comparisons across all windows of size Ω. Note that for each sub‐global extent, pairwise dissimilarities were only calculated for pairs of smaller cells that occur within a given larger cell (e.g., between Ψ = 0.25°‐equivalent cell grain, within Ω = 1° × 1°‐equivalent extent), while at the greatest extent (Ω = global) pairwise dissimilarity was calculated for any possible pair of smaller cells across the globe. Note that before this step, within‐century and within‐decade β diversity estimates (pairwise dissimilarity of any given pair of cells *i*) are averaged to enable temporally consistent comparisons, following the same steps described in the “Section 2.4.” Finally, we report dissimilarity change between each time‐period and the first time‐period of each time‐series (10th century and 1st decade of the 1700s, for the centurial and decadal time‐series, respectively; see “Section 2.4” for more details on how net‐change was calculated). All mean variability estimates (including mean, SE, SD, CIs, min, max, quantiles) are presented in Table S5.

**FIGURE 3:**
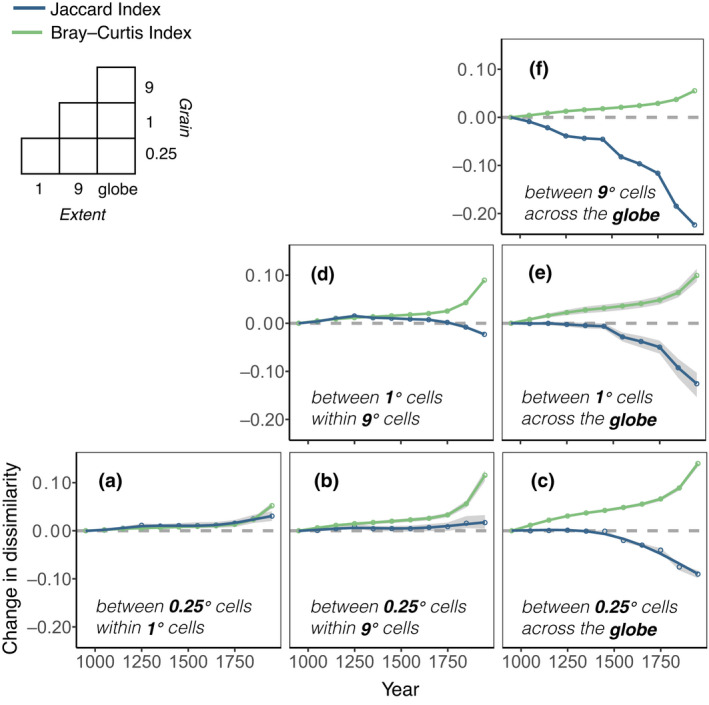
Temporal trends in spatial diversity of ecosystems. (a–f) Average total dissimilarity change between pairs of smaller cells (grain) within increasing larger cell areas (extent) as measured by the Jaccard index (ecosystem type presence‐absence dissimilarity—blue lines) and Bray–Curtis index (ecosystem type presence‐absence and area coverage dissimilarity—green lines) between each time‐period and the first time‐period (10th century) of the time‐series, plotted on the mid‐point of the century. Continuous lines are smoothing splines applied through the average estimates. Upper left‐side legend shows the different grains of analysis and extents considered. For (a, b, c, e) diversity change is characterized by the average dissimilarity change from 100 random draws (each draw ~1% of the full data set), and dark gray shading the range where 80% of all 100 draws means fall (upper and lower bounds are given by first and ninth deciles, respectively, at each time‐period). The decomposition of total dissimilarity into its components is shown in Figure S7 (centurial) and Figure S8 (decadal).

For certain combinations of grain and extent (grain of 0.25°‐equivalent at any extent and 1°‐equivalent at the global extent—Figure 3a,c,e) calculating pairwise dissimilarities for *all* possible pairs was computationally intractable. For these combinations, we randomly selected a subset of 1730 cells (∼1% of the full data set), made pairwise comparisons ($p_{k,i^{\Psi }}^{\Omega }$) between those cells, and then estimated $\bar{\beta \left(\Omega ,\Psi \right)}$. We repeated this exercise for 100 random draws, providing a mean (of the 100 draws) dissimilarity change and interdecile range (among the 100 draws). For sub‐global scales, we also conducted separate analysis for the two alternative ecosystem classifications, to test the robustness of our results to change in ecosystem categorization. The final number of pairwise comparisons for each combination of grain and extent are shown in Table S5. The interdecile range of all 100 different sample means (i.e., area where 80% of all means fall) is shown in both Figure 3 and Figures S9 and S10, where the upper and lower bounds are given by the first and ninth deciles of all 100 means across each time‐period.

Although the primary thematic resolution and spatial grain for analysis was nine ecosystems and 0.25°‐equivalent cells, respectively, we also evaluated whether the change of ecosystem diversity varied with scale and ecosystem categorization by conducting separate analysis using cells equivalent to the areas of 1°, 4°, 9° and 15° grid cells at the equator (Figures S2, S4 and S5) and the two alternative and more finely divided 61‐type and 126‐type classifications of ecosystem (see Section 2.2).

## 3 RESULTS AND DISCUSSION

### 3.1 Declines and persistence of “primary” ecosystems

As expected, the data show declining areas of primary forested and primary non‐forested land, with a higher rate of decline after 1700, and growing areas of multiple human‐dominated land uses (Figure S1). This conclusion holds across biomes, although some regions were heavily transformed prior to 1700 (Figure S1G,H). Surprisingly, the *frequencies* of areas (landscapes) that include at least some primary forested and primary non‐forested land remain largely unaltered. That is, most (97.4% for forests and 97.8% for non‐forested land) of the 0.25° cells where these ecosystem types were present in the 10th century still contained at least some area of the same ecosystem type in the 20th century (Figure S1; Table S1). This means that while we have seen substantial post‐1700 declines in areas of primary land cover, some amount of these ecosystem types still persist almost everywhere.

### 3.2 Diversity of ecosystem types

At the 0.25°‐equivalent cell resolution, all five indices showed significant and continuous increases since the 10th century, with a clear increase in rates from 1700 onwards, evident on both the centurial and decadal time scales (Figure 1). We judged relationships as significant when bootstrapping revealed no overlap in the bounds (defined by the interdecile range) of replicates between the beginning and end of the millennial time‐series. Mean global ecosystem richness increases are predominantly driven by a reduction in the number of cells containing one to four ecosystem types and increases in areas supporting five or more ecosystem types (Figure 1a inset). This is a consequence of the frequencies of different anthropogenic ecosystems (e.g., pastures, cropland) growing considerably faster than the frequencies of primary and secondary vegetation declined (Figure S1F). The net effect of modification has been to increase ecosystem diversity: an increased number, evenness, heterogeneity, and compositional entropy of ecosystem types.

This trend of increasing diversity at the 0.25°‐equivalent cell resolution changes after the mid‐20th century: the rate of increase slows for ecosystem richness, flattens for Shannon heterogeneity, shows a possible downturn (but shallower than the interdecile range) for evenness and Simpson heterogeneity, and reverses for Rao's quadratic entropy index (Figure 1). This is coincident with the “Great Acceleration” of the human population, technologies and associated impacts (Brolin & Kander, 2020; Steffen et al., 2015). Rao's index was the only metric to decline significantly (Figure 1j), reflecting the reduced biological distinctiveness of different anthropogenic ecosystem types (i.e., they often share species with one another; Newbold et al., 2015), whose cover increased during this period (Figure S1).

These temporal trends were replicated for most spatial resolutions of analysis, although some variation in the shape of the trends was observed after the mid‐20th century. In particular, Rao's index only reversed at sub‐regional scales (<4°‐equivalent cell resolution, <~197,000 km^2^ at the equator; Figure 1), while any tendencies for flattening or downturn in evenness and heterogeneity disappear. Ecosystem diversity continued to increase when coarser scaled grids were considered, for all four metrics, but typically at a reduced rate (Figure 1; gray lines). Despite some downturns, average ecosystem diversity for the 20th century remained higher than the averages of any preceding century for all metrics at all spatial scales considered (0.25°‐equivalent to 15°‐equivalent cells; Figures S4 and S5).

Trends were also robust to changes in ecosystem categorization, where we make different assumptions about whether we consider there to be 9 (Figure 1), 61 (only natural ecosystems vary between bioclimatic regions), or 126 ecosystem types (both natural and anthropogenic ecosystem types vary between bioclimatic regions; Figures S4 and S5). Varying ecosystem classification does not change the shapes of within‐grid cell temporal trends, with no influence on the conclusions in relation to spatial resolution for most metrics (the rank order of evenness metrics across scales in recent decades showed small changes; Figures S4 and S5).

Finally, our conclusions remain true after removing areas where the LUH2 data is known to have reduced levels of certainty. To evaluate this, we repeated the global analyses but excluded data for northern Africa and parts of western Asia (Figure S8; i.e., excluding regions of the world with sparse spatial information and where the LUH2 data set is known to have some allocation issues for primary and secondary vegetation). The overall pattern of ecosystem diversity change over time was retained for all five diversity metrics.

The pattern of increasing ecosystem diversity holds qualitatively for different regions of the world (IPBES sub‐regions and WWF biomes), albeit with geographic variation. At 0.25°‐equivalent resolution, Rao's quadratic entropy index, for example, revealed net diversity increases since the 10th century, rate increases from 1700 onward, and a mixture of slow‐downs and reversals in the 20th century across most regions and biomes of the world (Figure 2; see Figures S6 and S7 for other diversity metrics). Nearly all IPBES regions (16 out of 17) showed a net increase in accumulated ecosystem diversity using this metric between the 10th and 20th centuries, and a majority (13 out of 17) did so between 1700 and 2000, but with high interdecile variation (Figures S6 and S7). Divergent trajectories likely reflect the timing of different human impacts in different regions.

### 3.3 Spatial diversity changes

Our measures of β diversity tended to increase over time, although there were important differences in the results, depending on the grain (size of cells) and the extent (maximum distance between pairs of cells being compared) analysed. They also varied with the index of β diversity used. The Jaccard dissimilarity index, which measures differences in which ecosystem types are present (incidence) in different locations, revealed a pattern of relatively stable or slightly increasing differentiation over time at sub‐global scales (within ≤9°‐equivalent cells: ~997,000 km^2^ at the equator, or approximately the size of Egypt) but homogenization at a global extent (Figure 3; Figures S9–S11). The latter is consistent with some individual ecosystem types becoming present in more cells across the globe (e.g., at least small areas of croplands are found in large numbers of cells). This is consistent with narratives of biological homogenisation at a global scale, when considering that some species associated with particular land uses, such as urban areas, may have global distributions within this ecosystem type (Daru et al., 2021; Newbold et al., 2018). However, at within‐country scales (≤9°‐equivalent cells) homogenisation was not evident.

In contrast, the Bray–Curtis dissimilarity index, which incorporates the area of each ecosystem type as well as which ecosystem types are present, showed growing differentiation over time across all scales (grains and extents; Figure 3). Such differentiation was mostly driven by the areas of some ecosystem types declining and other ecosystems increasing between cells (balanced variation component of Bray‐Curtis; Figures S9 and S10). This means that, increasingly, some locations have high percentages and others low percentages of particular ecosystem types. There was a possible slight shift in rates of change of spatial differentiation in the mid‐20th century, but no reversals of previous trends (Figure S10). Using different numbers of ecosystems overall, the β diversity trends across time yielded similar overall conclusions, although the absolute values differed (Figure S11). Thus, when the area of each ecosystem type is taken into consideration, we see evidence of increased differentiation at all spatial scales, including global, rather than homogenisation.

### 3.4 Ecosystem change not loss

The changing diversity and distributions of ecosystems represent a major global change, of importance to regional and global‐scale ecosystem processes and the provision of services (IPBES, 2019). These changes underlie the CBD aspiration to achieve “no net loss” of ecosystems by 2030 (CBD, 2020). While some ecosystem types have indeed declined (mainly declining in area within a given landscape rather than completely eliminated), “lost” ecosystems have been replaced by a variety of anthropogenic ecosystems, increasing the numbers of ecosystem types per 0.25° landscape in most parts of the world (Figures 1 and 2), and also increasing spatial differentiation within most country‐sized regions (Figure 3). Many of these transformations are of great antiquity, reflecting the diversity of the peoples who inhabited them, making these parts of the planet less hospitable to some species but more so for others. In fact, ongoing conservation programmes commonly highlight the human and biodiversity value of cultural and indigenous landscapes in all six populated continents (Adom, 2016; Ens et al., 2016; Molnár & Berkes, 2018). Articulating all of these ecosystem changes as “loss” does not capture the full range of realities of the transformed Anthropocene world, and should be replaced by a narrative of ecosystem “change.” The losses are of major conservation importance, but so are the gains.

The replacement of primary ecosystems by anthropogenic ecosystems (land‐use cover types) that often support impoverished biotas can potentially result in a loss of local (e.g., per m^2^ or per ha) species richness (Newbold et al., 2015), and locations that today share anthropogenic ecosystem types may share increasing numbers of species (Daru et al., 2021; McGill et al., 2015). Anthropogenic ecosystems can also promote the establishment of already‐widespread species (Barnosky et al., 2011; Hiley et al., 2016; Hobbs et al., 2013), while more narrowly distributed native species decline (Newbold et al., 2018). Such conclusions have led to an overall narrative of ecosystem and biodiversity “decline and homogenisation.” However, the increasing diversity of ecosystem types that we observe could have the opposite effect at a landscape or regional scale (e.g., in 0.25°‐equivalent cells), given that ecosystem diversity is a major determinant of total species richness (Stein et al., 2014). After land‐use change, many native species survive in the remaining areas (fragments in some places) of original ecosystems, whereas additional colonising species establish in different semi‐natural and anthropogenic ecosystems (Desrochers et al., 2011). This contrast between plot‐scale species‐richness results (Newbold et al., 2015) and our landscape‐scale ecosystem diversity results may help explain why observed biodiversity changes are scale‐dependent (Jarzyna & Jetz, 2018; McGill et al., 2015). In conjunction with the transfer of species between regions, these results can also help explain why regional‐scale analyses, particularly of plant diversity, typically show increases in the number of species present per region (Ellis et al., 2012; Sax & Gaines, 2003). At the landscape scale (between the local and regional), heterogeneity created by land‐use can also help to maintain or increase plant diversity (Vellend et al., 2021). Nonetheless, depending on the spatial scale, locations and periods vary in whether they show increases or declines in numbers of species (Capinha et al., 2015; Ellis et al., 2012; McGill et al., 2015; Pimm et al., 2014; Sax & Gaines, 2003; Thomas, 2013, 2015). These contrasting trends come together within the Rao index results (Figures 1 and 2), which incorporate the diversity of ecosystems but “down‐weight” ecosystem types that often support overlapping biotas (e.g., cropland and pasture). Rao's index shows strongly increasing diversity over the full period, but it is also the one metric to exhibit a clear decline in diversity in the second half of the 20th century. Therefore, our results are consistent with the recent literature on species (but not ecosystem per se) homogenisation associated with land‐ use changes over this period. Yet the millennium‐long effects of human‐caused land‐use changes have been the opposite.

As a caveat, it is important to emphasise that our results relate to the diversity of ecosystem types, as defined, with the Rao index also providing an expectation concerning changing patterns of species richness over time at the landscape‐ to country‐sized spatial scales studied. That do not relate to changes in the specific identities of species associated with those ecosystems (including through human transport, and the impacts of climate on species compositions within ecosystems) or to numbers of individual species that are threatened by land‐use change (e.g., in contexts where the most of an original ecosystem type has been removed). Nonetheless, the total diversity of ecosystems and associated numbers of species per landscape or country would appear to have increased over the full millennium period, suggesting some potential for ecological resilience.

In conclusion, we observe net ecosystem diversification across the globe over the last millennium of human transformations of the Earth's ecosystems, and spatial differentiation at sub‐global scales—prior to the mid‐20th century. Although the global story of biodiversity change involves the loss and decline of certain ecosystem types, the full story is more complex and interesting, involving gains and increases in other ecosystem types, and increased ecosystem diversity at most spatial and temporal scales. Hence, and despite the unambiguous reductions in the extent of primary ecosystems, it seems appropriate to temper language emphasizing only habitat and ecosystem “loss” with descriptions of ecosystem “change.”

## AUTHOR CONTRIBUTIONS

Conceptualization: I.S.M., M.D., M.V., and C.D.T.; Methodology: I.S.M., M.D., M.V., and C.D.T.; Software: I.S.M.; Formal Analysis, I.S.M.; Visualization: I.S.M.; Writing—Original Draft: I.S.M. and C.D.T.; Writing—Review and Editing: I.S.M., M.D., M.V., and C.D.T.; Supervision: M.D., M.V., and C.D.T.; Funding Acquisition: C.D.T.

## CONFLICT OF INTEREST

Authors declare no competing interests.

## Supporting information


Appendix S1

Table S1‐S5

## Data Availability

Supporting data (including summary tables) and all R codes used for data processing and analysis are archived online at Zenodo (https://doi.org/10.5281/zenodo.6802742). The original land‐use data used is published and publicly available at https://luh.umd.edu/data.shtml.
