# Supplementary material for: A millennium of increasing diversity of ecosystems until the mid‐20th century
Source: Glob Chang Biol. 2022 Jul 22;28(20):5945–55. doi: 10.1111/gcb.16335 (PMC9543278; doi:10.1111/gcb.16335)
Supplement: Supplementary file 1 — Appendix S1 [file GCB-28-5945-s001.pdf]

Supporting Information

for

A millennium of increasing diversity of ecosystems until the mid-20th century

Inês S. Martins[1,2,3], Maria Dornelas[2,3], Mark Vellend[2,4], Chris D. Thomas[1,2]

[1]Department of Biology, University of York; Wentworth Way, York YO10 5DD, UK.

[2]Leverhulme Centre for Anthropocene Biodiversity, Berrick Saul Second Floor, University of York; York YO10 5DD, UK.

[3]Centre for Biological Diversity, School of Biology, University of St Andrews; St Andrews, KY16 9TF, Scotland, UK.

[4]Département de Biologie, Université de Sherbrooke; 2500 boulevard de l'Université, Sherbrooke, QC, Canada.

This file contains **Supplementary Information**, which includes eleven Supplementary Figures and five Supplementary Tables (only legends; tables are uploaded separately).

Supplementary Figures

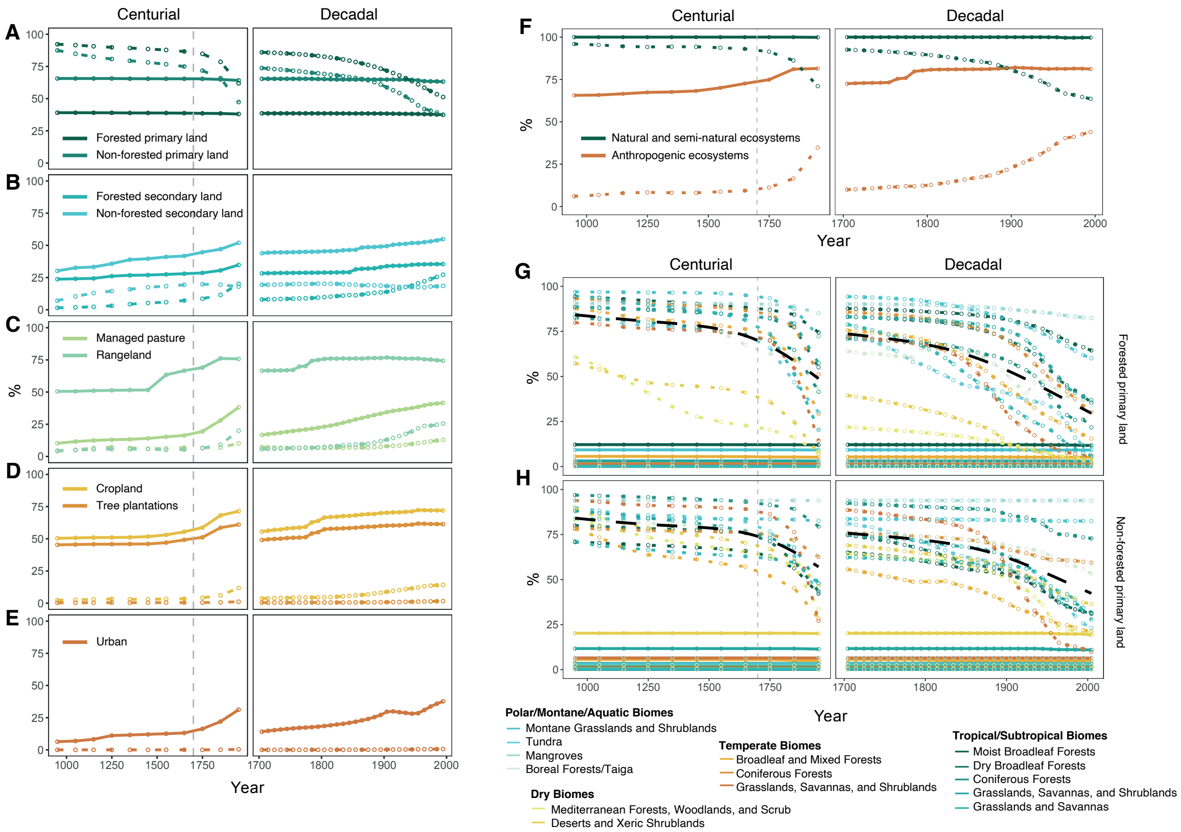

Figure S1. Global trends in land-use change for the nine ecosystem types, from 900 to 2000. Plot shows average presence (i.e. proportion of all cells where the ecosystem is present - solid line) and coverage (i.e. average proportion of the grid cell occupied by a given ecosystem type across all cells where that ecosystem is present - dashed line) of **(A)** primary land, **(B)** secondary land, **(C)** rangeland and pasture, **(D)** agricultural, and **(E)** urban ecosystems. **(F)** shows average presence and coverage for 2 macro-classes: natural and semi-natural ecosystems (primary land, secondary land) and anthropogenic ecosystems (rangeland and pasture, agricultural, and urban ecosystems). **(G)** and **(H)** show, respectively, the breakdown of forested and non-forested primary land across the 14 WWF Biome, where colours represent different regions and black the global trends. Note, that points on the left-hand graphs represents the spatial average values across all equal-area cells (equivalent to 0.25º x 0.25º cell area at the equator) found over a 100-year period plotted on the mid-point of the century, while the right-hand graphs show the decadal averages (from 1700 to 2000) plotted on the mid-point of the decade. For better visualization, average estimates for a given ecosystem (A-E) or region (F-G), are connected through a line.

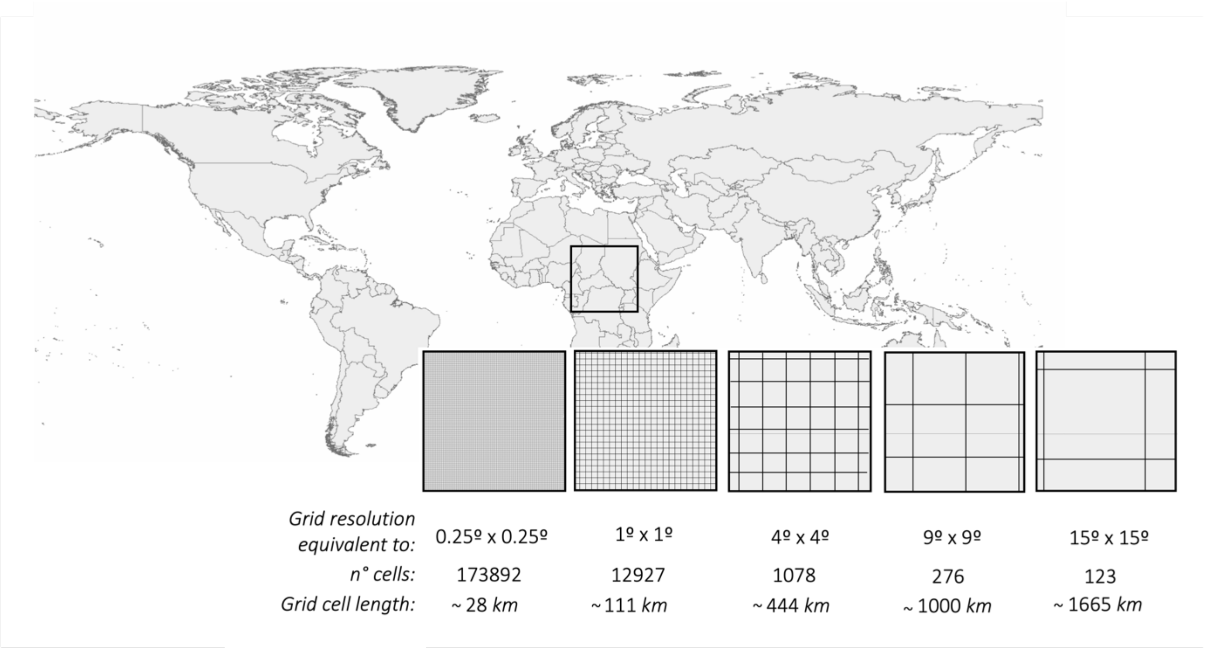

Figure S2. Graphical illustration of the study grains of analysis. The primary spatial grain for analysis was 0.25º-equivalent cell resolution, but scale-dependence was evaluated by conducting separate analysis using grain size cells of increasing size (1º-, 4º-, 9º- and 15º-equivalent cell resolution).

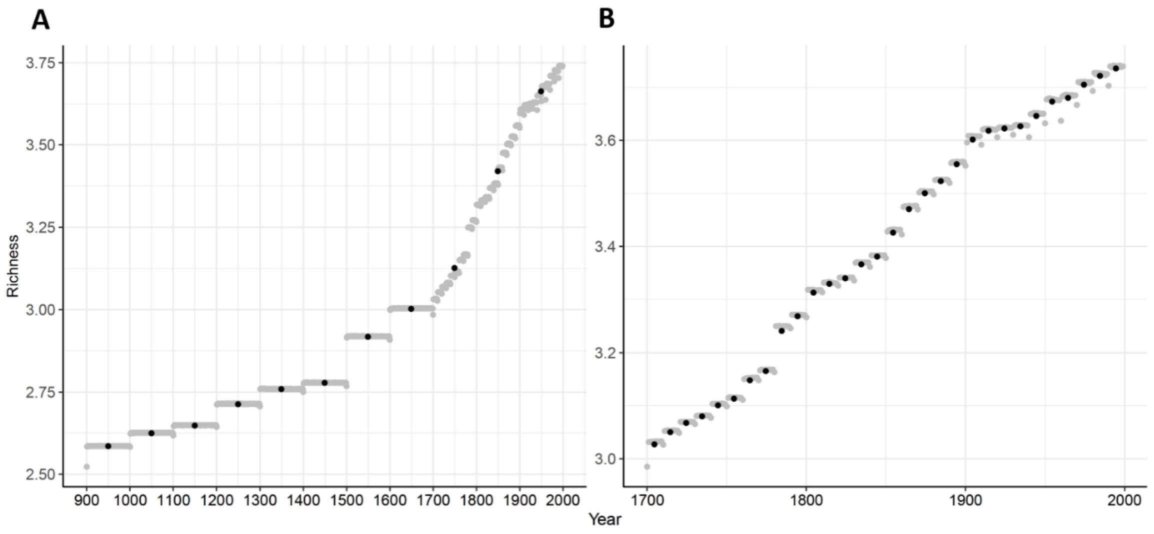

Figure S3. LUH2 input data temporal resolution. The original land-use data behind the LUH2 model had an underlying temporal resolution of every 100 years from 900 to 1700 and every 10 years from 1700 to 2000. An algorithm was used by the LUH2 developers to transform century- and decadal-long estimates to annual estimates based on a set of assumptions (*6*). In our analysis, we re-aggregated to **(A)** centurial and **(B)** decadal temporal resolutions (e.g., for ecosystem richness, shown here; black points), because individual year values (grey points) are not independent of one another. For centurial analyses, we also calculated mean values for 100-year intervals after 1700.

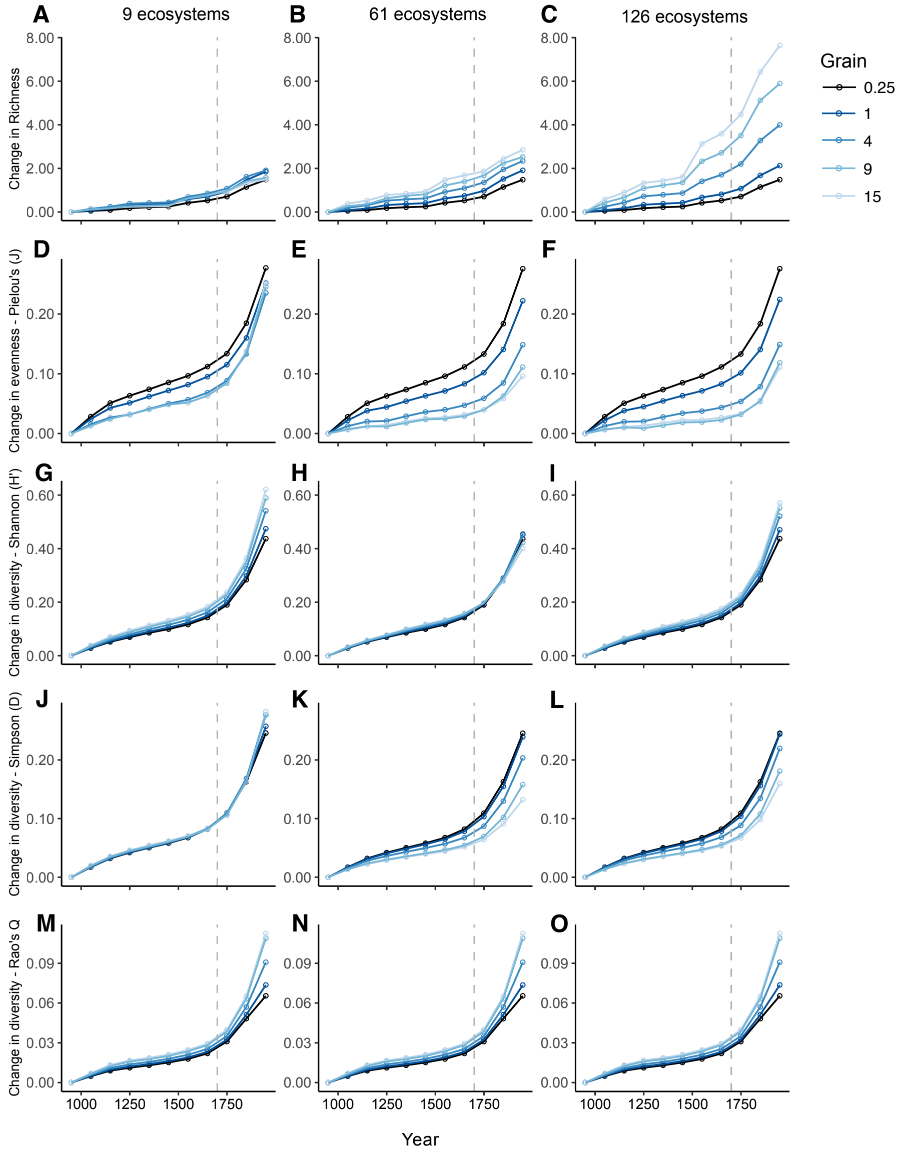

Figure S4. **Net change in local ecosystem diversity (α diversity) from 900 to 2000 (centurial trends) using different spatial resolutions of analysis and categorisations of ecosystems.** Plots show net change in local ecosystem diversity when we consider there to be 9 **(A, D, G, J, M;** original classification**)**, 61 (**B, E, H, K, N**; alternative classification 1) or 126 (**C, F, I, L, O)**; alternative classification 2) ecosystem types for five different grains of analysis (0.25º, 1º, 4º, 9º and 15º-equivalent cell resolution). Each point on the graph represents the spatial averaged change between each time-period and the first time-period (10th century) of the centurial time-series, plotted on the mid-point of the century, for a given metric: **(A, B, C)** within-cell ecosystem richness (change in mean numbers of ecosystem types per cell), **(D, E, F)** Pielou's evenness, **(G, H, I)** Shannon diversity index, **(J, K, L)** Simpson diversity index, and **(M, N, O)** Rao's quadratic entropy index. All continuous lines are smoothing splines applied through the average estimates for a given grain.

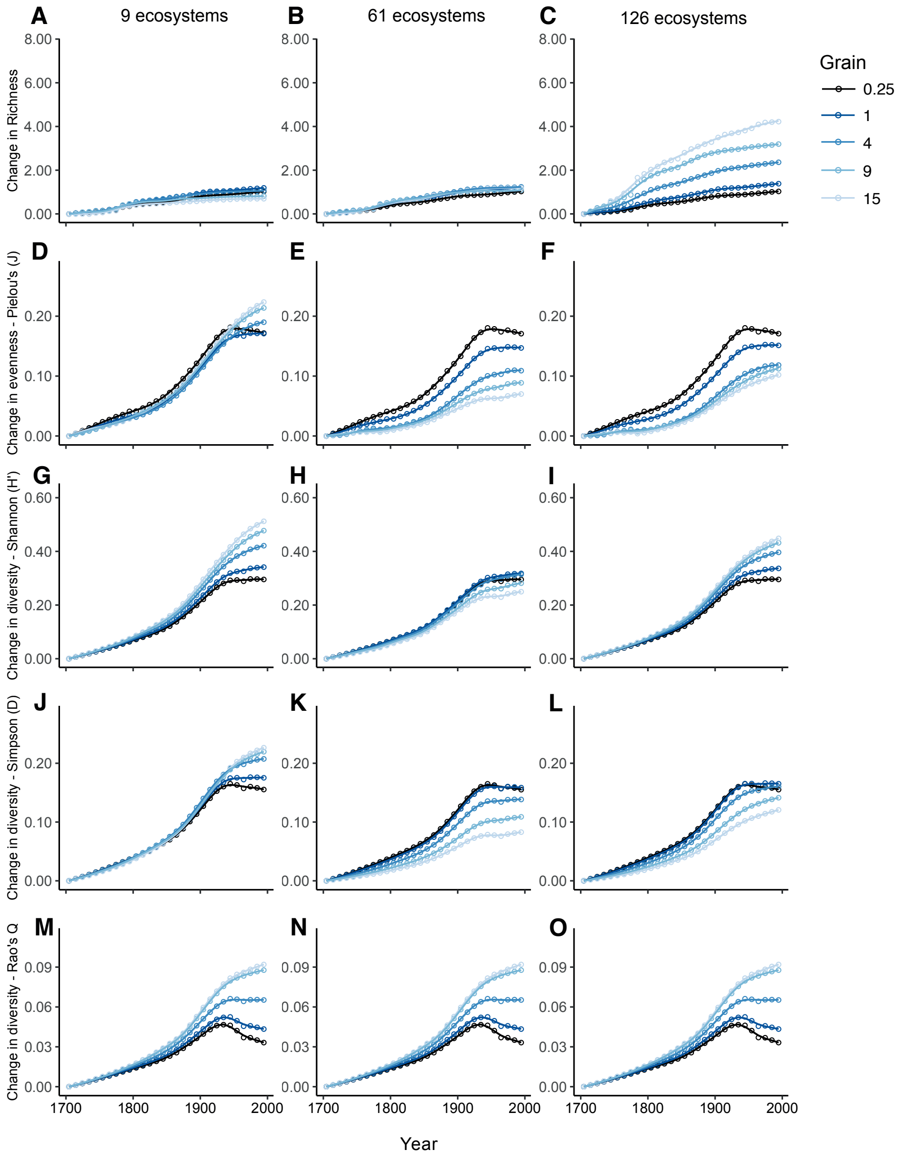

Figure S5. Same as **Figure S4**, but showing net change in local ecosystem diversity (α diversity) from 1700 to 2000 (decadal trends), where each point on the graph represents the spatial averaged change values between each time-period and the first time-period (1st decade of 1700) of the decadal time-series, plotted on the mid-point of the decade.

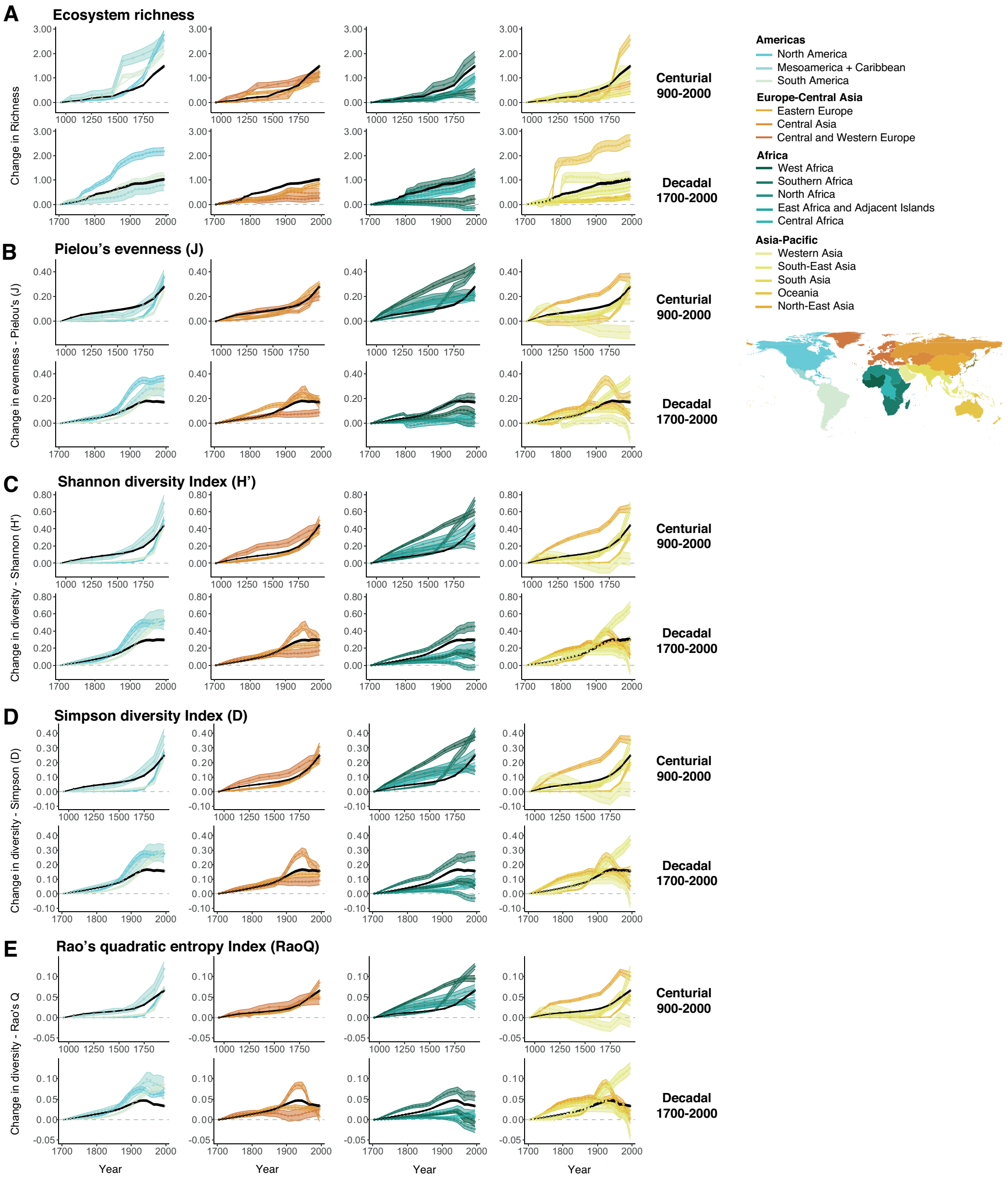

Figure S6. Ecosystem diversity trends (α diversity) for each IPBES sub-region. Average regional changes of **(A)** within-sample ecosystem richness (change in mean numbers of ecosystem types per cell), **(B)** Pielou's evenness, **(C)** Shannon diversity index, **(D)** Simpson diversity index, and **(E)** Rao's quadratic entropy index across cells (0.25° x 0.25°-equivalent grid cells) found over a 100yr, plotted on the mid-point of the century, and 10yr period, plotted on the mid-point of the decade and shown relative to the baseline of the time-series (10th century and 1st decade of 1700, respectively). All continuous lines are smoothing splines applied through the average estimates for a given region; Shading represents the range where 80% of all 1000 draws means fall (upper and lower bounds are given by first and ninth deciles, respectively, at each time-period). Colours represent different regions; black lines show the global trends.

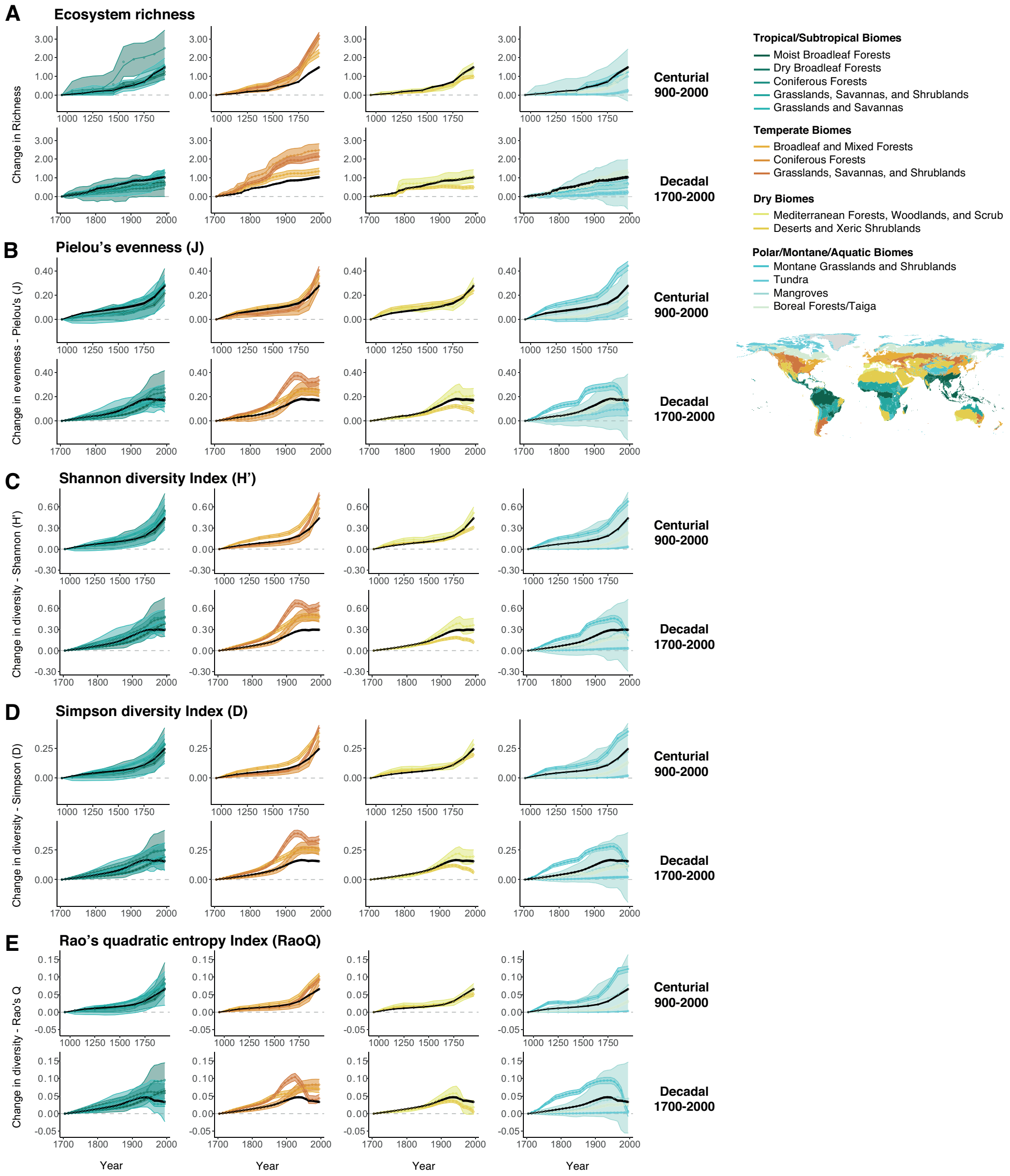

Figure S7. Same as **Figure S6,** but showing average change within each WWF Biome.

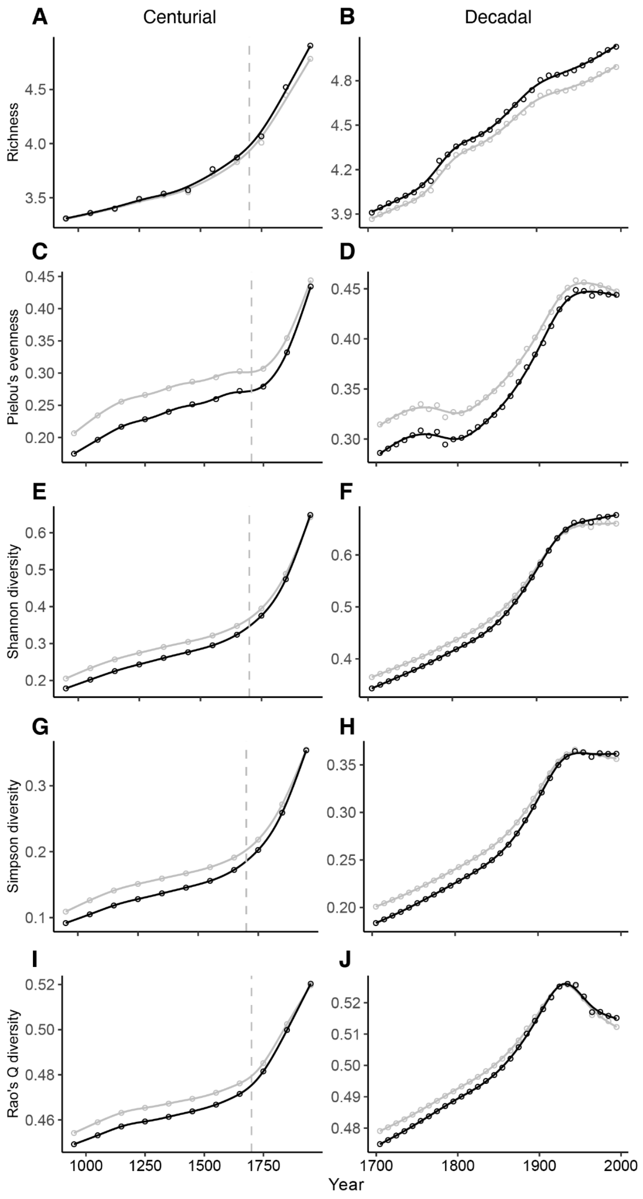

Figure S8. The impact of North Africa and Western Asia cells on global estimates of ecosystem coverage. Plots shows **(A-B)** within-cell ecosystem richness (mean numbers of ecosystem types per cell), **(C-D)** Pielou's evenness, **(E-F)** Shannon diversity index, **(G-H),** Simpson diversity index, **(I-J)** and Rao's quadratic entropy index when considering all cells (173,892 cells; grey line) and when excluding North Africa and Western Asia cells (158,445 cells; black line). Where grey lines cannot be seen, they lie beneath the black lines, indicating minimal effect of North African and Western Asian cells. The differences relate to the balance of primary and secondary non-forested land, which is a true reflection of the predominance of dryland ecosystems in these regions.

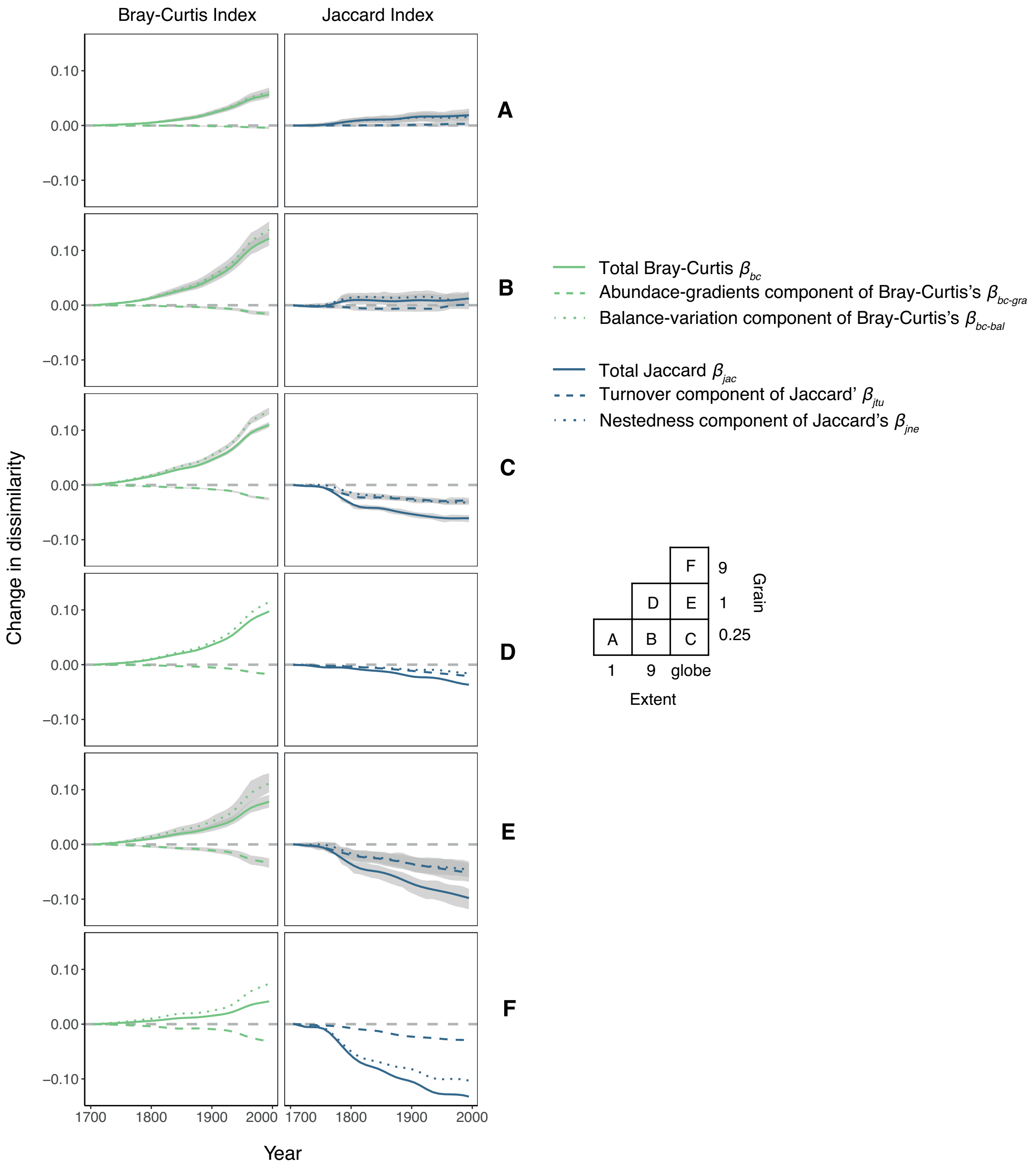

Figure S9. Decomposition of spatial β diversity centurial patterns. Plots show the different components of dissimilarity trends shown in Figure 3. The Bray-Curtis index (β_{bc}) is split into balanced variation (β_{bc-bal}) and abundance gradient (β_{bc-gra}) components, and the Jaccard index (β_{jac}) into turnover (β_{jtu}) and nestedness (β_{jne}) components. For (A, B, C, E) diversity change is characterized by the average dissimilarity change from 100 random draws (each draw ~1% of the full dataset), and dark grey represents the range where 80% of all 100 draws means fall (upper and lower bounds are given by first and ninth deciles, respectively, at each time-period).

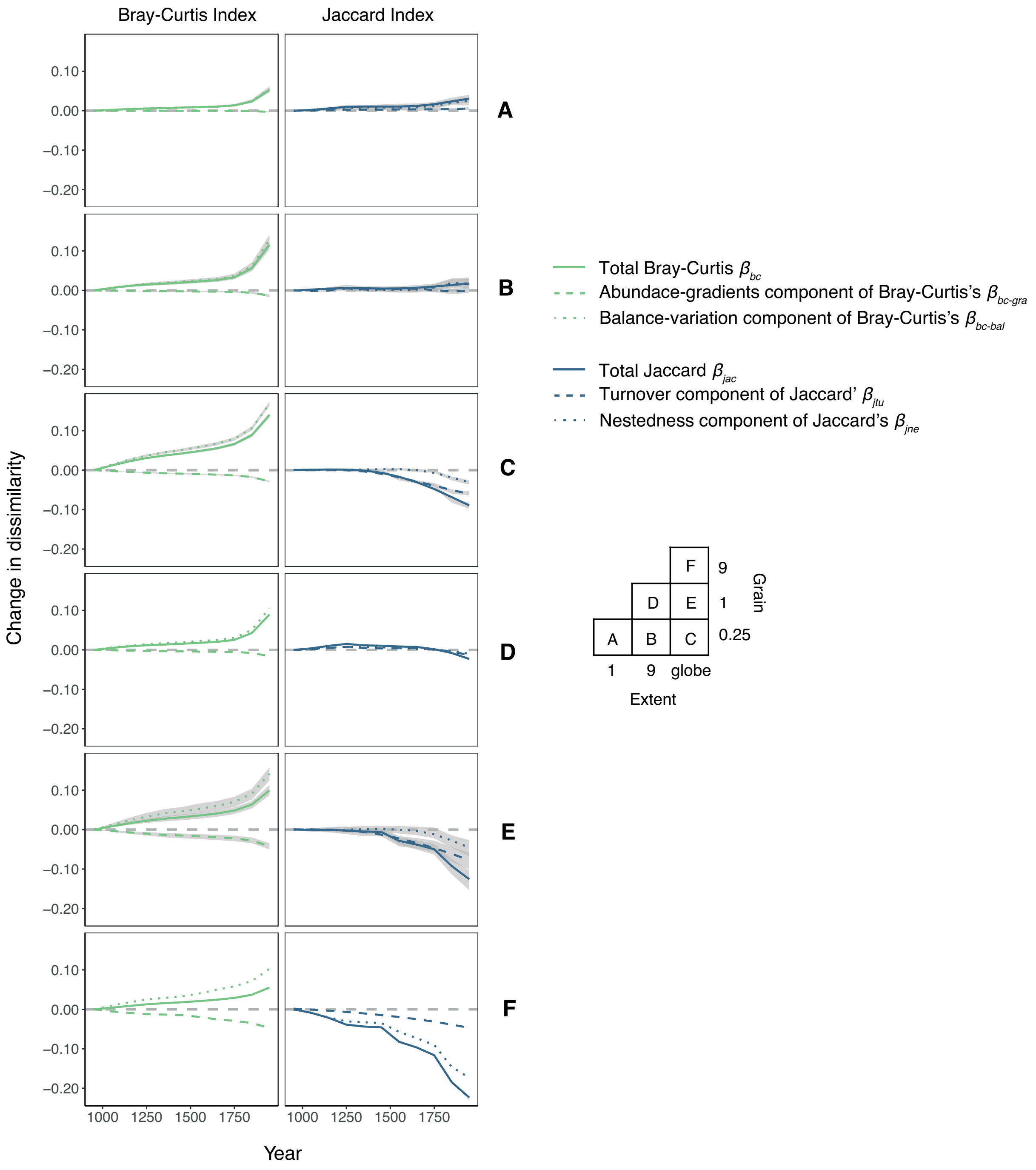

Figure S10. Same as **Figure S9,** but showing decadal trends.

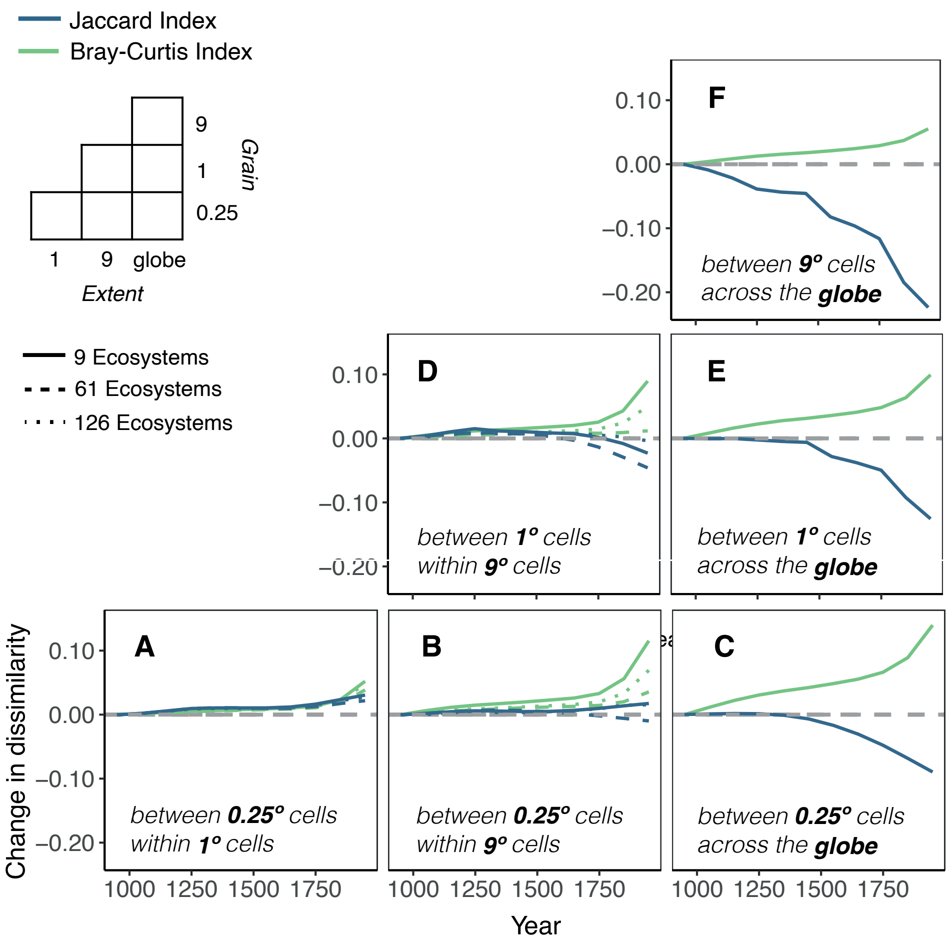

Figure S11. Temporal trends in spatial β diversity of ecosystems using distinct ecosystem categorizations. (**A-F**) Average total dissimilarity change between pairs of smaller cells (grain) within increasing larger cell areas (extent) as measured by the Jaccard index (ecosystem type presence-absence dissimilarity - blue lines) and Bray-Curtis index (ecosystem type presence-absence and area coverage dissimilarity - green lines) between each time-period and the first time-period (10th century) of the time-series, plotted on the mid-point of the century. Continuous lines are smoothing splines applied through the average estimates. Upper left-side legend shows the different grains of analysis and extents considered. For (A, B, C, E) diversity change is characterized by the average dissimilarity change from 100 random draws (each draw ~1% of the full dataset). For sub-global extents (A, B, D) diversity change was also measured using two alternative categorisations of ecosystems: 61 ecosystems (dotted lines) and 126 ecosystems (dashed lines).

Supplementary Tables (uploaded separately)

General notes: Across all supplementary tables, the column "*variable*" always refers to the mid-point of the decade (when "*leg*" = Decadal) or century (when "*leg*" = Centurial) considered. The column "*scale*" and "*region*" indicate the scales of analysis (global or regional) and the individual region, respectively, for which the ecosystem diversity estimates were calculated. Column "*type*" refers to absolute estimates (when "*type*" =abs) or within-cell net-change (when "*type*" =rel), and column "*class*" the ecosystem classification considered: 9_eco=9 ecosystems, 61_eco=61 ecosystems and 126_eco=126 ecosystems. In Table S2-S4 column "*metric*" indicates the α diversity metric: R-within-cell ecosystem richness, J-Pielou's evenness, H-Shannon diversity index, D-Simpson diversity index, Q-Rao's quadratic entropy index; while in Table S5 it indicates the β diversity metric: J-Jaccard dissimilarity index, B-Bray-Curtis dissimilarity index.

Table S1- Average change in the presence and coverage of the different ecosystem types from 900-2000. Includes all data shown in Figure S1. Column "*n_all*" shown number of cells considered, "*sum_lu*" the total area of a given ecosystem type across all cells, "*frac_n_lu*" the proportion of cells where the a given ecosystem is present, "*frac_wlu0*" the average proportion of the grid cell occupied by a given ecosystem type across all cells and "*frac_wluw0*" the average proportion of the grid cell occupied by a given ecosystem type across the cells where that ecosystem is present. Column "*version*" refers to change across (V1) all cells or (V2) excluding North Africa and Western Asia cells.

Table S2 - Global and regional (IPBES sub-regions and WWF Biomes) changes in local ecosystem diversity (α diversity). Includes all global and regional data shown in Figure 1 and 2. Column "*wlu_mean*" shows the spatially-average mean estimates across all equal-area cells of a given grain (column "*grid*") for a given metric (column "*metric*"), region (column "region"), and time-period (colum "*variable*"). Column "*type*" refers to absolute estimates (when "*type*" =abs) or within-cell net-change (when "*type*" =rel). Other global and regional variability estimates (se, sd, CIs, min, max, quantiles) are also included.

Table S3 - Individual 1000 draws used to visualize the variability in the global means. Includes all estimates (mean, se, sd, CIs, min, max, quantiles). Column "*sim*" refers to the individual draw numbers (from 1-1000) and "*n*" number of cells in the sample.

Table S4 - Mean variability estimates across the 1000 individual draws. Includes all (mean, se, sd, CIs, min, max, quantiles) global (Table S3) and regional estimates. Column "*sim*" refers to the individual draw numbers (from 1-1000) and "*n*" number of draws.

Table S5 - Temporal trends in spatial $\boldsymbol{\beta}$ diversity of ecosystems. Includes all data shown in Figure 3. Column "*n_pairs*" refers to the total (when "*n_sim*" =1) or average across draws (when "*n_sim*" =100) number of pairwise comparisons for each combination of grain and extent. Column "*code*" refers to the individual combinations of grain*extend shown in Figure 3.
